# The impact of Blackboard Collaborate breakout groups on the cognitive achievement of physical education teaching styles during the COVID-19 pandemic

**DOI:** 10.1371/journal.pone.0279921

**Published:** 2023-01-06

**Authors:** Ahmed Hassan Rakha

**Affiliations:** Faculty of Physical Education for (Men–Girls), Department of Curriculum and Teaching Methods of Physical Education, Port-Said University, Port-Said, Egypt; Zayed University, UNITED ARAB EMIRATES

## Abstract

The rapid spread of COVID-19 has forced schools and universities to close. Globally, education systems face unprecedented challenges, and learning management systems (LMS) are the only solution. The current study aimed to investigate the effectiveness of a Blackboard collaborative breakout group on the cognitive achievement of physical education teaching styles. The quasi-experimental method involved creating two groups: one experimental and one control, with the experimental group using Blackboard collaborative breakout groups and the control group relying exclusively on online lectures and continuing with the same method without breakout groups. The study sample consisted of 40 students who were randomly assigned and divided equally into the two groups. Based on the research sample, homogeneity within the group and equivalence between groups in terms of age, Grade Point Average (GPA), and high intelligence test (IQ) were evaluated. The results showed that the experimental group’s cognitive achievement was superior to that of the control group. Therefore, the design of the learning process enhances student collaboration, participation, and reinforcement. Additionally, the experimental group retained the learning outcomes for a month after the cessation of all teaching and learning processes. To conclude, giving a lecture using webinar tools such as Blackboard Collaborate Ultra does not necessarily mean achieving the intended educational goals. As a result, it is necessary to look for ways to integrate active learning strategies, such as collaborative learning, to enhance student involvement in distance learning.

## Introduction

To stem the spread of COVID-19, public health measures have resulted in the closure of schools and universities. Globally, education systems face unprecedented challenges. Government agencies are collaborating with international organizations, private sector partners, and members of civil society to deliver distance education using a variety of technologies. In this way, the educational process is maintained and all students learn. To achieve student engagement and curriculum goals, the development and implementation of distance learning strategies must be urgently explored and expanded to avoid exacerbating existing educational and social disparities [1]. As a result of the COVID-19 pandemic in higher education, universities used distance learning exclusively for some courses, either synchronously or asynchronously, as well as blended learning for others, especially practical courses, to protect students’ rights to learn and develop academic and non-academic skills [2].

During the COVID-19 pandemic, learning management systems (LMSs) were the only solution for continuing education. Foreman [3] describes this as a web-based software application that helps organizations manage training events, self-paced training courses, and blended learning programs. This, in turn, manages learning content, student engagement, assessment tools, learning progress reports, and student activities through the automation of processes. The majority of universities around the world use LMSs, either open-source like Moodle or WebCT, or closed-source like A Tutor or Blackboard. Blackboard is one of the most popular LMSs worldwide; it was developed by the American educational technology company "Blackboard Inc." It is designed to support technology-based learning, blended learning, or online learning because it contains both synchronous and asynchronous tools [4]. It is one of the most widely used LMSs in Saudi universities.

Blackboard includes asynchronous tools such as course descriptions, course content, assignments, course messages, discussion boards, blogs and wikis, pools, tests, etc. A full-grade center is also included. Moreover, the company has developed Blackboard apps for both teachers and students which can be used on Android and IOS mobile devices [5,6]. Blackboard Collaborate Ultra is integrated with the synchronous attendance feature in virtual classrooms. It is a web-based system with several tools that make traditional educational activities easier. These tools include the presentation window, which enables group video conferencing sessions, the chat system, which lets students communicate privately while still in class and respond to questions from teachers, and an automated polling mechanism for gathering feedback. Sessions should be captured and made available to all students after each lesson and throughout the whole course, while a breakout room facilitates group and collaborative work among students by randomly assigning them to teams and setting up separate rooms so that they can converse with various people during each class meeting. Students converse with members of their group most often by speaking to one another [5,7].

With the COVID-19 pandemic, social distancing has become a necessity. This has had an effect on people’s social and academic lives. Even in the classroom, cooperative learning has become nearly impossible, even though students must work with their peers and support one another. It is clear from all of the literature that student collaboration benefits learning [8–11]. This is especially true for courses that demand a high level of critical thinking and problem-solving abilities [12]. There is more to online teaching than creating a library of learning materials or broadcasting a lecture [13]. Creating a fun, enjoyable, engaging, collaborative, comfortable, and engaging learning environment is an essential part of an online teacher’s job [14].

Stephen [15] reports that webinar platforms were effective educational tools during the COVID-19 pandemic, but the educational outcomes were insufficient. In spite of the fact that webinars can provide continuous, engaging, and customized learning experiences. Dasgupta et al. [16] concluded that webinars cannot replace face-to-face education. Additionally, webinars need to overcome a number of technical challenges and resource constraints, as well as increase the participation of learners in the learning process. According to Peper et al. [17], students are unable to concentrate and remain unresponsive when attending synchronous online classes. Furthermore, students have described feelings of isolation, anxiety, and depression when they are online.

According to the foregoing, there is a gap between the functionality of webinar tools and the results achieved in increasing student participation and efficiency in distance education. Also, despite the importance of active learning strategies, particularly collaborative learning, it is imperative to provide mechanisms that enable them to be used when teaching online. Accordingly, the significance of the current study lies in the ability to design learning to incorporate collaborative learning techniques into online teaching. This is done by utilizing the breakout groups feature in Blackboard Collaborate Ultra.

Therefore, the current study aimed to investigate the effectiveness of a Blackboard collaborative breakout group on the cognitive achievement of physical education teaching styles. The spectrum of teaching styles in physical education theory by Mosston and Ashworth [18] is an important included in the Teaching methods of physical education course at the seventh level in the physical education and kinesiology program at Qassim University, because it takes up half of the total contact hours and necessitates that students develop knowledge, skills, and competences about each teaching style and how to integrate them. This course, which has 1 theoretical and 1 practical credit hour, is one of the fundamental requirements for students to enroll in field training. Blackboard Collaborate Ultra was selected as one of the webinar tools because it is an official app approved by Qassim University and integrated with the university’s academic system.

## Theoretical framework

### Active learning

Active learning is any learning strategy in which students are actively involved. During active learning, students engage in meaningful learning activities and reflect on their experiences [19]. In place of lectures, active learners solve problems and create knowledge through student-centered exercises [20]. Active learning involves the student actively instead of passively [21]. Active learning encompasses a wide range of instructional strategies that teachers might implement in the classroom. Collaborative learning, cooperative learning, and problem-based learning are three of its subcategories [22]. All these strategies aim to engage students in the learning process [23]. Collaborative learning is a structured form of group work in which students pursue common goals while being assessed individually [24]. Based on historical trends, linguistic roots, and practical applications, Davidson and Major [25] explained the differences between cooperation and collaborative learning. A distinction between them can be made from an applied perspective in light of the current study, in which Panitz [26] defines cooperative learning as a set of processes that help students interact to accomplish a specific objective or develop a specific product. The content is usually specific, and the goal is usually achieved. It is more directive and closely controlled by the teacher than collaborative learning. Thus, the essential difference between the two terms is that the primary approach to cooperative learning is teacher-centered, whereas collaborative learning is more student-centered and enables students to interact with each other as they decide on participation and effort. Students are responsible for navigating and searching for information and resources. Both interpretations emphasize the importance of student interactions rather than individual learning. As a general rule, Davidson and Major [25] found that the development of higher-order thinking skills (HOTS) is positively correlated for the two terms. To ensure students work as a team, a variety of strategies have been developed. A few cooperative learning techniques have been reviewed by Davidson and Major [25], including Think-Pair-Share [27], Three-Step Interview, Timed Pair Share [28], and Jigsaw [29]. Kitchen and McDougall [30] argue that one of the main distinguishing features separating collaborative learning from cooperative learning is that its core objective is to make students take responsibility for working together. This requires a transfer of responsibility from the teacher to the students. In addition, by working together to achieve a common goal, students build knowledge through interaction with each other.

### Cooperative learning theory

A cooperative learning environment occurs when students work together as part of a group to complete a task [31]. A constructivist cognitive development theory and a social Interdependence theory are the basis of cooperative learning theory [32]. With its roots in Piaget’s cognitive development and Vygotsky’s sociocultural theory, constructivist cognitive development theory has emerged as the dominant paradigm for pedagogical development in recent decades. The idea that knowledge is not transferred from teachers to students but is constructed in the minds of students is fundamental to the application of constructivism in the classroom. The focus of knowledge in this case is not on how the teacher imparts knowledge to the students, but on how they can construct knowledge for themselves. Teachers are shaped by constructivism because it represents a shift away from behaviorism-based education to cognitive theory-based education [33]. Social Interdependence Theory identifies two types of interdependence between individuals: positive interdependence, which is characterized by individuals striving to achieve mutual goals, and negative interdependence, which occurs when individuals obstruct the goals of one another. As a result, the teacher strives to foster positive social interdependence among students so that they can achieve the desired knowledge structure on their own [34]. A cooperative learning approach is distinguished from most instructional approaches by its social interdependence theory. A teacher can structure cooperative learning most effectively, modify it for various educational contexts, and apply it to a wide range of issues including achievement, and ethnic integration when they understand social interdependence [35].

And from the above, It is possible to engage in cooperative learning in three different ways: formally, informally, or in a cooperative base group [36]. In formal cooperative learning groups, students can complete any course requirement during a single class period. An instructor introduces the lesson, divides the class into two to five groups, provides supplies for the activity, and assigns duties. Teachers observe students’ collaboration methodically. Teachers intervene if students lack understanding of academic tasks or have problems collaborating. Upon completion of the project, the teacher assesses each student’s academic progress and asks the group to evaluate their performance. The goal of collaboration is to bring students who care about one another’s success together. Informal cooperative learning groups form impromptu teams during lectures, demonstrations, and movies. These groups draw attention to the material, create a learning environment, set expectations, ensure students understand the content, and conclude the lesson. It can last for a few minutes or an entire class period. Cooperative base groups (lasting one semester or a year) provide support, assistance, and encouragement to members so that each can advance academically and socially [35,37].

### Cognitive load Theory (CLT)

According to the CLT, working memory capacity is one of the most important indicators of effective learning outcomes in a classroom setting. Working memory has a limited capacity. Consequently, complex material, which consists of numerous interconnected elements of information, will be difficult to comprehend. The reason for this is that working memory is limited. Therefore, learners should maintain these elements in working memory and link them to understanding the material. As a result of this cognitive load, working memory capacity is frequently exceeded (i.e., it is overloaded). Thus, successful learning requires a working memory that can handle what is required. Hence, learning success depends on cognitive processes during learning, which put cognitive demands on working memory [38,39].

Sweller [38] distinguished between useful cognitive load (intrinsic, germane) and unnecessary cognitive load (extraneous). Intrinsic load is the difficulty of a task resulting from how many information elements and their interrelationships must be learned. In other words, it refers to the amount of working memory devoted to basic processing necessary for understanding the subject matter. A Germane cognitive load adds to the intrinsic load, consuming additional working memory by constructing schemas and storing them in long-term memory. As a final point, extraneous cognitive load occupies the working memory capacity through activities that are not related to understanding the subject matter, building schemas, or automating procedures [38–40]. Extraneous load is heavily influenced by how content is presented. Poor presenting designs can impede learning because they increase extraneous cognitive load. In order to be successful at learning, none of the three categories of cognitive load should put too much strain on the working memory. According to cognitive load theory, an excessive amount of external and internal strain can lead to working memory overflow [39].

### Instructional Design (ID) models

Educators and designers can incorporate technology into education using a variety of models. In order to create an effective learning design process, developers can use these models as a framework to guide their work, enabling them to produce an effective learning program which focuses on the student rather than the teacher. Koper [41] defined ID as the instructional process within a learning unit (e.g., a course, a lesson, or another type of learning activity). As one of its primary characteristics, one of the key features of ID is the way it represents both the learning and support activities carried out by both the teacher and the learner within the context of a learning unit. In order to promote learning, ID strives to organize events in a systematic manner. A number of steps are involved in the ID process, including the analysis of learners and contexts, the development of outcomes, methods, and evaluation instruments, the creation of educational resources, assessing learners’ performance, and evaluating the identification process as a whole [42].

**Fig 1:**
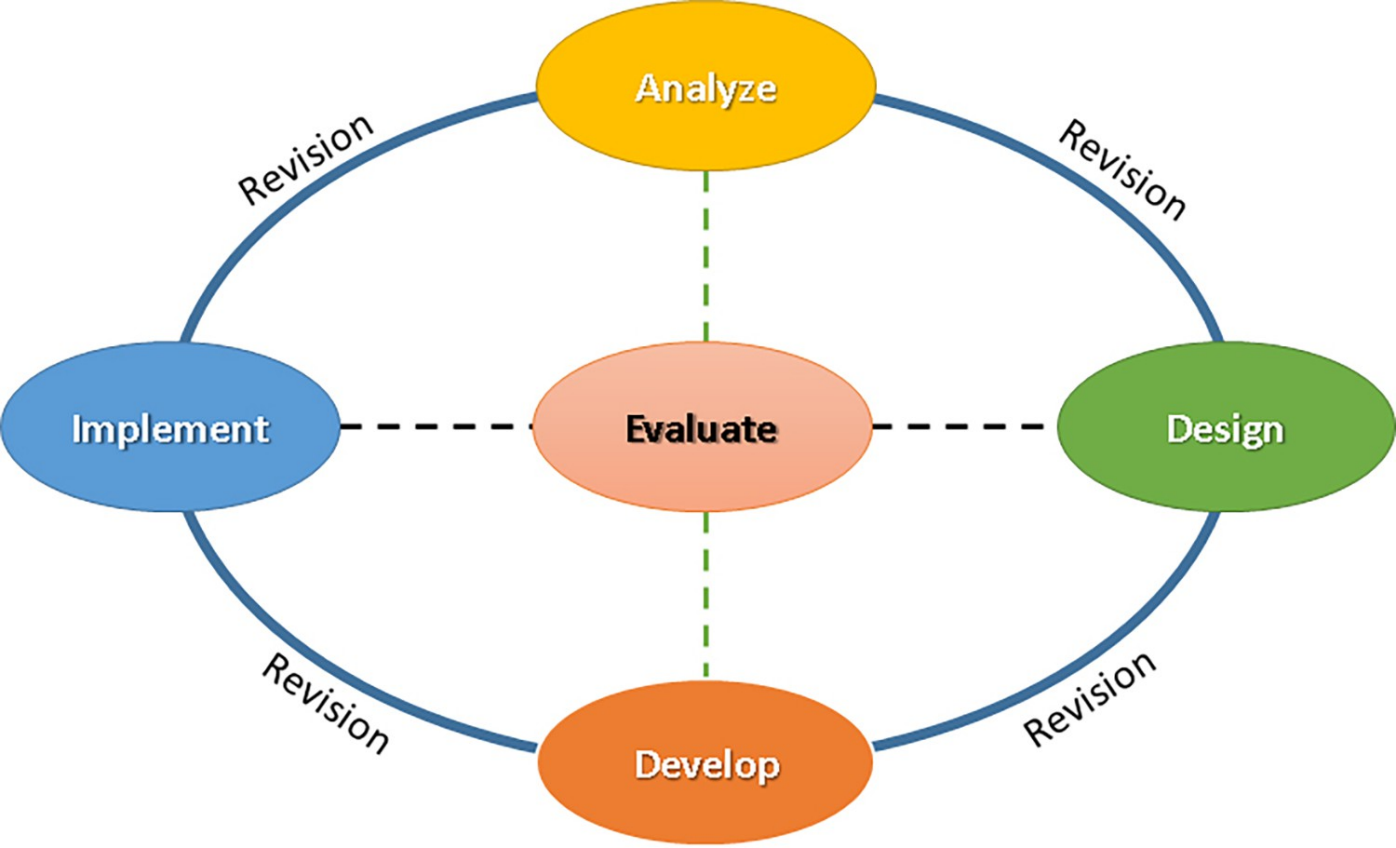
ADDIE model [43].

Among the most widely used and well-known models of ID are ADDIE, DDD-E, ASSURE, Morrison, Ross, and Kemp, as well as Smith and Ragan. An ADDIE model was used in the current study as shown in (Fig 1).

**Fig 2:**
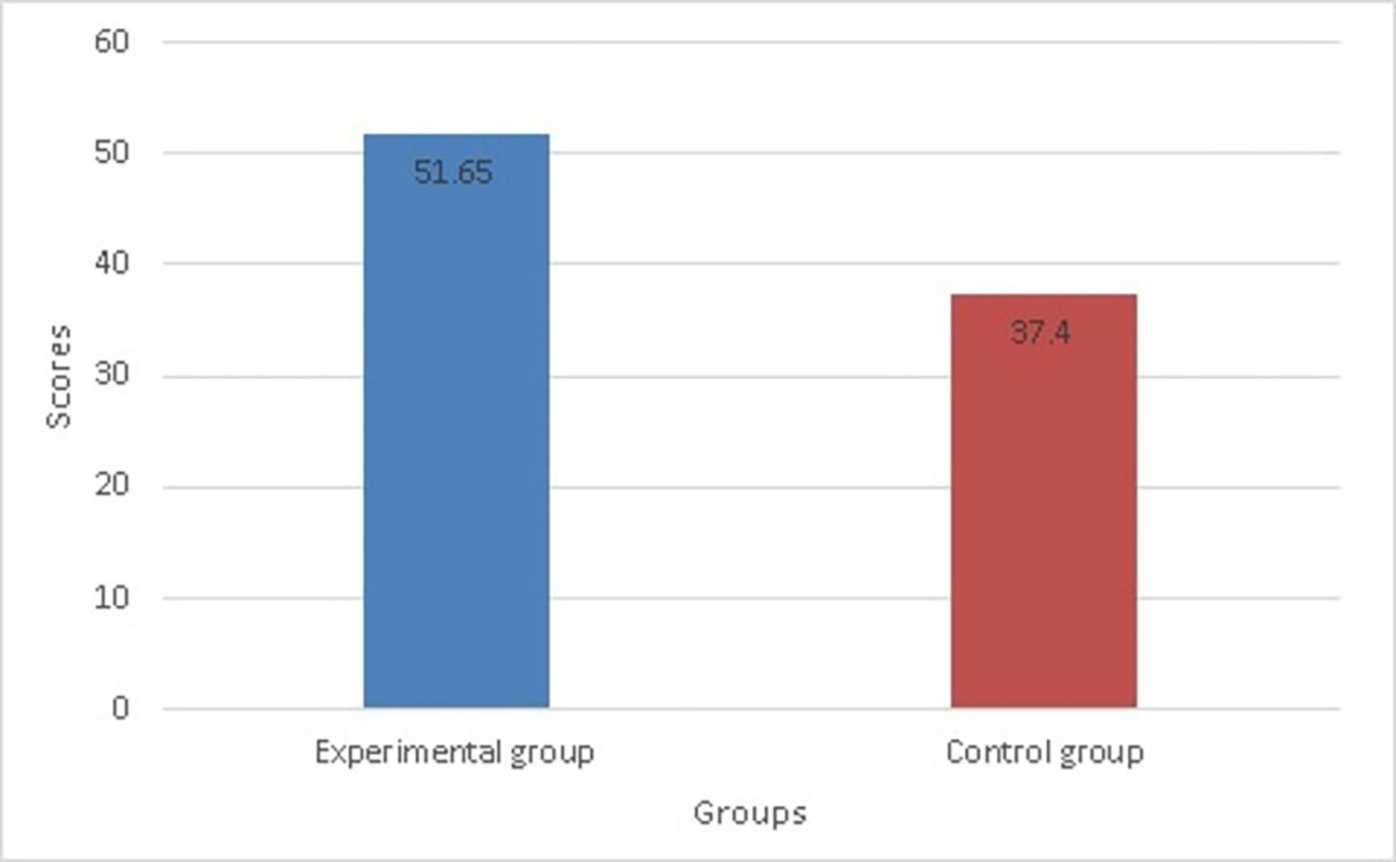
The significant differences between the two groups.

The ADDIE model is a popular ID model for creating instructional materials. These five steps are outlined as follows, as seen in Fig 2: (1) Analyze the learners, the topics they will be learning, and the expected learning outcomes. (2) Design, which includes objectives for learning, content, teaching strategies, delivery methods, and learning activities. (3) Development, such as the creation of multimedia interactive systems. (4) Implementation, which entails delivering instructional materials to learners. (5) Evaluation is a crucial part of the model. It ensures that the learning process always stays on track by giving access to various stages depending on the situation and ensuring that the learning process produces the desired outcomes [43,44].

## Materials and methods

### Ethics statement

The study was conducted in accordance with the ethical standards of the Scientific Research Committee at the Department of Physical Education and Kinesiology, College of Education, Qassim University (Approval No. 11444182022). To obtain informed consent from study participants, the author developed an online Google form based on the COVID-19 pandemic. Several aspects of the study were addressed in the online form, including voluntary participation, withdrawal rights, objectives, importance, procedures, and confidentiality. The author ends the form by asking the participants, "Would you like to participate in the study?". In response to this question, you will select Agree or Disagree to indicate whether or not you agree to participate.

### Design

The quasi-experimental method involved creating two groups: one experimental and one control, with the experimental group using Blackboard collaborative breakout groups and the control group relying exclusively on online lectures and continuing with the same method without breakout groups. To determine the extent of the continuity of the impact of Blackboard collaborative breakout groups, the post-test was administered to the two groups immediately following the completion of the teaching and learning processes, while the follow-up test was administered after no further teaching and learning processes had taken place for a month.

### The hypotheses

1. *Ha*: In the post-test, in the level of cognitive achievement of physical education teaching styles, there are statistically significant differences between the means of the control group (Without collaborated breakout groups) and the experimental group (With collaborated breakout groups) in favor of the experimental group.
2. *H*_*0*_: For the experimental group, there are no statistically significant differences between the post-test and follow-up test in regard to cognitive achievement level.
3. *H*_*0*_: For the control group, there are no statistically significant differences between the post-test and follow-up test in regard to cognitive achievement level.

### Study population and sample

The participants were randomly selected from seventh-level students who were enrolled in the Physical Education and Kinesiology Program for men at Qassim University in the second semester of the 2020/2021 academic year and who were registered for the Physical Education Teaching Methods course. There were 69 students in total. The study sample consisted of 40 students who were randomly assigned and divided equally into two groups: control and experimental. Based on the research sample, homogeneity within the group and equivalence between groups in terms of age, Grade Point Average (GPA), and high intelligence test (IQ) [45] were evaluated.

According to Table 1, there was a normal distribution for the control group. The skewness values ranged from -1.22 (*SE* = 0.55) to 0.25 (*SE* = 0.55).

**Table 1:** Descriptive statistics of the control group (*n* = 20).

Variables	Lowest value	Highest value	M	SD	Skew	SE
Age (y)	21	23	22.20	0.62	-0.12	0.55
IQ (Score)	45	66	54.25	8.62	0.25	0.55
University GPA	3	4.59	4.06	0.38	-1.22	0.55

According to Table 2, there was a normal distribution for the experimental group. The skewness values ranged from -0.55 (*SE* = 0.55) to 0.09 (*SE* = 0.55) [46].

**Table 2:** Descriptive statistics of the experimental group (*n* = 20).

Variables	Lowest value	Highest value	M	SD	Skew	SE
Age (y)	21	23	22.35	0.67	-0.55	0.55
IQ (Score)	45	69	57.65	7.15	-0.01	0.55
University GPA	2.83	4.74	3.88	0.61	0.09	0.55

Table 3 shows the results of Levene’s test for equality of variances. This test identifies whether the variances of the two groups are significantly different. The F-value of Levene’s test for age was 0.97, while for IQ it was 2.99, and the *P*-value was greater than 0.05, being 0.33 and 0.09, respectively. Thus, the independent samples t-test can be used to indicate the differences between the two groups for both variables. Meanwhile, the F-value for the GPA variable was 8.40 and the P-value was less than 0.05, amounting to 0.01. Therefore, the Mann-Whitney *U* test was used to calculate the significance of the differences between the two groups in that variable.

**Table 3:** Independent samples compare means.

Variables	Levene’s Test for Equality of Variances		Mean Differences	*t*	*P*
	*F*	*P*			
Age (y)	0.97	0.33	0.15	0.74	0.47
IQ (Score)	2.99	0.09	3.40	1.36	0.18
			Mann-Whitney U		
University GPA	8.40	0.01	162.50		0.31

According to an independent samples t-test, it is clear that there are no statistically significant differences between the experimental and control groups in the variables of age and IQ level, where the value of *t* (38) = 1.36 and *t* (38) = 0.74, respectively. Also, the p-value was greater than 0.05 (*p*>.05).

According to the Mann-Whitney U test, there were no statistically significant differences between the two groups in the University GPA, where the value of *U* (*n* = 20) = 162.50, *Z* = 1.01, *p* > .05. Therefore, the control and experimental groups are statistically equivalent for those variables.

### Data collection tools and equipment

#### The high IQ test

The IQ of university students was evaluated in Arabic [45]. The 42 questions in the test, which range in difficulty, cover a variety of mental functions. The test has been utilized in several studies with samples similar to the sample for the current study, including [47–49]; its validity coefficient was 0.69 and its reliability coefficient was 0.84.

#### Students’ cognitive achievement test

To achieve the research aim, the researcher designed a cognitive achievement test for students in the teaching physical education styles topic as follows:

1- Prepare a table of specification (TOS)

Frequently, Bloom’s taxonomy is used to describe behavior goals students should be able to accomplish upon completing their education. These are categorized into three domains: cognitive, psychomotor, and affective [50]. Based on complexity, the cognitive domain was divided into six levels: knowledge, comprehension, application, analysis, synthesis, and evaluation [51]. Bloom’s taxonomy was revised in 2001 and is now known as Bloom’s revised taxonomy. One of the most significant changes was the change from noun to verb forms for the six main categories. Moreover, "synthesis" was moved to the top of Bloom’s revised taxonomy as "creating". Krathwohl [52] defined the six levels as remembering, understanding, applying, analyzing, evaluating, and creating. Accordingly, the TOS in this study was based on that. As Gronlund [53] notes, a TOS, also called a blueprint, helps educators align learning outcomes, content, assessment, and the relative weights of cognitive-behavioral goals to achieve test balance. According to Wolming and Wikström [54], a well-designed TOS increases the validity of instructors’ evaluations. It assists in determining the topic and goal categories’ relative weights. Additionally, the following formulae are used to determine the number of topic questions and their scores:

-
$The\;relative\;weight\;of\;the\;topic\;\%=\frac{Teaching\;hours\;or\;days\;allocated\;to\;a\;particular\;topic}{Amount\;of\;time\;allotted\;to\;teaching\;the\;course’s\;topics}\times 100$

-
$The\;Relative\;weight\;of\;the\;learning\;outcomes\;category\;\%=\frac{The\;number\;of\;objectives\;in\;the\;category\;}{The\;Total\;Learning\;Outcomes\;in\;the\;course}\times 100$

-
Foreachtopic,Thenumberofitemsinthelearningoutcomescategory=Thetotalnumberofitems×Therelativeweightofthetopic×TherelativeweightoftheLearningOutcomescategory

-
Foreachtopic,ThescoreofLearningoutcomescategory=Testfinalscore×Therelativeweightofthetopic×TheRelativeweightoftheLearningOutcomescategory

The cognitive achievement test specification is shown in Table 4.

**Table 4:** Table of specification (TOS).

Topics	Items & scores	Learning Outcomes (LOs)						Total number of items	Total number of items adjusted	Test final score	Relative weight of the topics (24 hours) %
		Remembering	Understanding	Applying	Analyzing	Evaluating	Creating				
		9 LOs	4 LOs	6 LOs	2 LOs	2 LOs	1 LOs				
An overview of the spectrum (2 hours)	Items	0.94	0.42	0.63	0.21	0.21	0.10	2.5	3	5	8.33
	Scores	1.88	0.83	1.25	0.42	0.42	0.21				
The anatomy of any teaching style (2 hours)	Items	0.94	0.42	0.63	0.21	0.21	0.10	2.5	3	5	8.33
	Scores	1.88	0.83	1.25	0.42	0.42	0.21				
The cluster of styles A–E (8 hours)	Items	3.75	1.67	2.5	0.83	0.83	0.42	10	10	20	33.33
	Scores	7.5	3.33	5	1.67	1.67	0.83				
The cluster of styles F–G (8 hours)	Items	3.75	1.67	2.5	0.83	0.83	0.42	10	10	20	33.33
	Scores	7.5	3.33	5	1.67	1.67	0.83				
Mixing two or more styles (4 hours)	Items	1.88	0.83	1.25	0.42	0.42	0.21	5	4	10	16.68
	Scores	3.75	1.67	2.5	0.83	0.83	0.42				
Total number of items		11.25	5	7.5	2.5	2.5	1.25	30	30		
Test final score		22.5	10	15	5	5	2.5			60	
Total number of items adjusted		11	5	7	3	3	1	30			
Test final score adjusted		22	10	15	5	5	3			60	
Relative weight of the learning outcomes (24 LOs) %		37.50	16.67	25.00	8.33	4.17	8.33				100%

2- Formulating the test items

Test items were initially formulated using different formats, such as multiple choice, pairing, and true-false, covering the topics and measuring multiple and gradual levels of thinking from memory to creativity, as outlined in the TOS.

3- Content validity

The first step in developing the test was to consult five experts in physical education curriculum and methods to ensure that the items were suitable for the purpose and that the vocabulary was scientifically sound. They recommended a few items [55].

4- Difficulty index and discrimination index

The item difficulty index is a measure of the percentage of students who correctly answered the item. It ranges between 0% and 100%, with a higher value denoting an easier element. In general, P-values greater than 0.90 are easy to understand and may not be worth testing. If a P-value is lower than 0.20, it indicates that the item may be difficult and should be reviewed for potentially confusing language or content that needs to be re-taught. Items with moderate difficulty should be preferred over items with high difficulty [56,57]. Gregory [56] concluded that the item’s difficulty index is a useful tool for determining items that must be changed or omitted. The ideal difficulty level for an item is around 0.50, which falls between 0.30 and 0.70. The difficulty index was calculated using the following equation: $P=\frac{R}{N}$

*P* = difficulty index

*R* = number of examinees who get that item correct

*N* = total number of examinees [58]

The discrimination index measures how well an item distinguishes between high- and low-scoring examinees. It is denoted by *(d)*. In general, the upper and lower bands refer to the highest and lowest scores from 10% to 33% of the examinees. When the overall score distribution is normally distributed, the best comparison is between the highest 27% and the lowest 27%. When total test scores are distributed flatter than a normal curve, the optimal percent is closer to 33%. The following equation is used to calculate it: $d=\frac{U-L}{N}$

*U* = number of examinees in the upper range who answered the item correctly

*L* = number of examinees in the lower range who answered the item correctly

*N* = total number of examinees in the upper or lower range [56]

Discrimination index values range from -1.00 to +1.00. Negative discrimination items are rejected. In most academic achievement tests, items with discrimination indexes above 0.20 are generally acceptable [56]. According to Bichi [58], *d* can be interpreted as follows:

- *d* ≥ 0.40 Item is functioning quite satisfactorily.
- 0.30 ≥ *d* ≥ 0.39 Good item; little or no revision is required.
- 0.20 ≥ *d* ≥ 0.29 The item is marginal and needs revision.
- *d*≥ 0.19 Poor items; should be eliminated or completely revised.

On Sunday, March 28, 2021, [28] students who had finished studying the research topics and who were not included in the basic sample took the cognitive exam in its final form for the calculation of the test’s difficulty and discrimination index. The difficulty and discrimination index for the test items are shown in Table 5.

**Table 5:** Difficulty and discrimination index of test items.

Items	*P*	*d*	Items	*P*	*d*
***1***	0.3	0.61	***16***	0.3	0.54
***2***	0.4	0.68	***17***	0.5	0.68
***3***	0.5	0.57	***18***	0.3	0.54
***4***	0.3	0.61	***19***	0.5	0.64
***5***	0.3	0.68	***20***	0.4	0.61
***6***	0.3	0.57	***21***	0.6	0.71
***7***	0.4	0.64	***22***	0.2	0.68
***8***	0.3	0.50	***23***	0.2	0.75
***9***	0.3	0.64	***24***	0.2	0.75
***10***	0.6	0.64	***25***	0.3	0.61
***11***	0.4	0.61	***26***	0.7	0.71
***12***	0.4	0.71	***27***	0.5	0.68
***13***	0.4	0.61	***28***	0.3	0.64
***14***	0.6	0.71	***29***	0.1	0.75
***15***	0.3	0.71	***30***	0.3	0.75

5- Reliability

Cronbach’s alpha was used to calculate the reliability of the test; the result was 0.78, which indicates a high-reliability index. (Scores greater than 0.7 indicate good reliability [59]. S1 Appendix in the supplemental materials provides the final version of the cognitive achievement test of the spectrum of physical education teaching styles.

## The educational program using blackboard collaborate breakout groups

Through the following steps, ADDIE learning design was used to design learning on the Blackboard collaborate breakout groups platform:

1- Analysis: This stage involved determining the general objectives of the program, the characteristics of the students, and the educational activities.
  - The general objective is to improve students’ cognitive achievement of "the spectrum of physical education teaching styles".
  - Student characteristics: This study sample ranged in age from 21 to 23 years old. According to Spano [60], students in this age range can set and achieve goals, express ideas, maintain a sense of their role in life, demonstrate work ethics, take individual responsibility, and demonstrate cognitive development.
  - There are two types of educational activities: those performed by the teacher and those performed by the student. During the teacher’s instruction, students learn how to use Blackboard Collaborate (joining the class, using breakout groups, submitting assignments and tests, and communicating with other students and the teacher). In the control group, students interact with the Blackboard collaborative platform, communicate with their teacher and colleagues through audio and video, and follow the teacher’s presentation. For the experimental group, task sheets explain the formation and use of subgroups, as well as how to interact with each other. After the collaborative work groups have completed their tasks, the tasks are sent to the teacher on time and displayed in the main room.
2- Design: In this stage, behavioral objectives are set, a teaching strategy is defined, an assessment strategy is formed, and task sheets are designed for experimental groups to collaborate on.
  - The formulation of learning outcomes: In accordance with Bloom [50], 24 learning outcomes are formulated and divided into six cognitive levels.
  - Teaching strategy: With the control group, an online lecture was conducted using the Blackboard platform. Experiment groups used breakout-group tools in the Blackboard collaborative system and task sheets to reinforce collaborative learning.
  - Assessment strategy: As specified in the TOS for the test, an online cognitive test was designed to measure the topics under study and the cognitive levels.
  - Developing task sheets for the experimental group to use during breakout groups, containing instructions, learning outcomes, tasks, and performance times. As Mosston and Ashworth [18] point out, task sheets allow students to participate actively in the task, which reduces repetitive explanations by teachers and helps students learn to follow written instructions. Task sheets increase efficiency and productivity at work, and reduce repetitive explanations by teachers. S2 Appendix in the supplemental materials provides an example task sheet for organizing experimental breakout groups.
  - Time framework: According to the Physical Education Teaching Methods course credit hours (CR), one lecture was given per week to each experimental and control group, with two theoretical CRs for each lecture; one CR equaled 50 contact minutes. Over a five-week period, each experimental and control group received five lectures on the topics.
3- Development: Blackboard Collaborate Ultra was selected as one of the webinar tools because it is an official app approved by Qassim University and integrated with the university’s academic system. The educational resources (e-books, presentations) were downloaded using the Blackboard Collaborate platform and were presented to the students, allowing them to ask and discuss questions through the platform, in addition to the breakout groups’ tool that was unique to the experimental group.
4- Implementation: Over a five-week period, between 31/1/2021 and 3/3/2021, the main experiment was conducted on Sundays for the control group and Wednesdays for the experimental group. In the experimental group, the Blackboard Collaborate system was utilized with breakout groups for collaborative activities, while in the control group, the system was utilized in a traditional way without any collaborative activities. To assess cognitive achievement, each group took a post-test on 7/3/2021. One month after the discontinuation of the teaching and learning processes, a follow-up measurement was performed.

## Statistical analysis

IBM SPSS Statistics for Windows (2017; version 25; IBM Corp, Armonk, NY, USA) was used for the following statistical analyses: frequencies, percentage (%), mean (*M*), test standard division (*SD*), Levene’s Test (*F*), Kolmogorov-Smirnov (*D*), Cronbach’s alpha (*α*), skewness coefficient, Mann-Whitney (*U*), paired samples T-test, and independent samples T-test.

## Results

### First hypothesis results

*H*_*A*_: There were statistically significant differences between the control and experimental groups in the post-measurement of the level of cognitive achievement of physical education teaching styles, which favored the experimental group.

Table 6 shows that the F-value for Levene’s test was 0.73 and the P-value was greater than .05; thus, the variances were not significantly different from one another. Therefore, we can use the *t* value and degrees of freedom. For the level of cognitive achievement of physical education teaching style scores, Table 6 and (Fig 2) show that the 20 students who used Blackboard Collaborate with breakout groups to learn (*M* = 51.65, *SD* = 2.92) and the 20 students in the control group who used Blackboard virtual classroom without breakout groups (*M* = 37.40, *SD* = 3.51) indicated statistically significant differences, with a t value of *t* (38) = 13.93 and a *p*-value of ≤.05. Consequently, the experimental group demonstrated significantly better cognitive achievement of physical education teaching styles than the control group as shown in (Fig 2). Therefore, this null hypothesis is rejected.

**Table 6:** Significant differences between the two groups using independent sample T-tests regarding their level of cognitive achievement (n1 = n2 = 20).

Variables	Experimental Group	Control Group	Levene’s Test for Equality of Variances		Mean Differences	*df* *t*	*p*
	*M* *SD*	*M* *SD*	*F*	*P*			
Cognitive achievement (60 degree)	51.65 2.92	37.40 3.51	.73	.40	14.25	38 13.93	.00

### Second hypothesis results

*H*_*0*_: There were no statistically significant differences between the post-measurement and the follow-up measurement for the experimental group in the level of cognitive achievement of teaching physical education styles.

**Fig 3:**
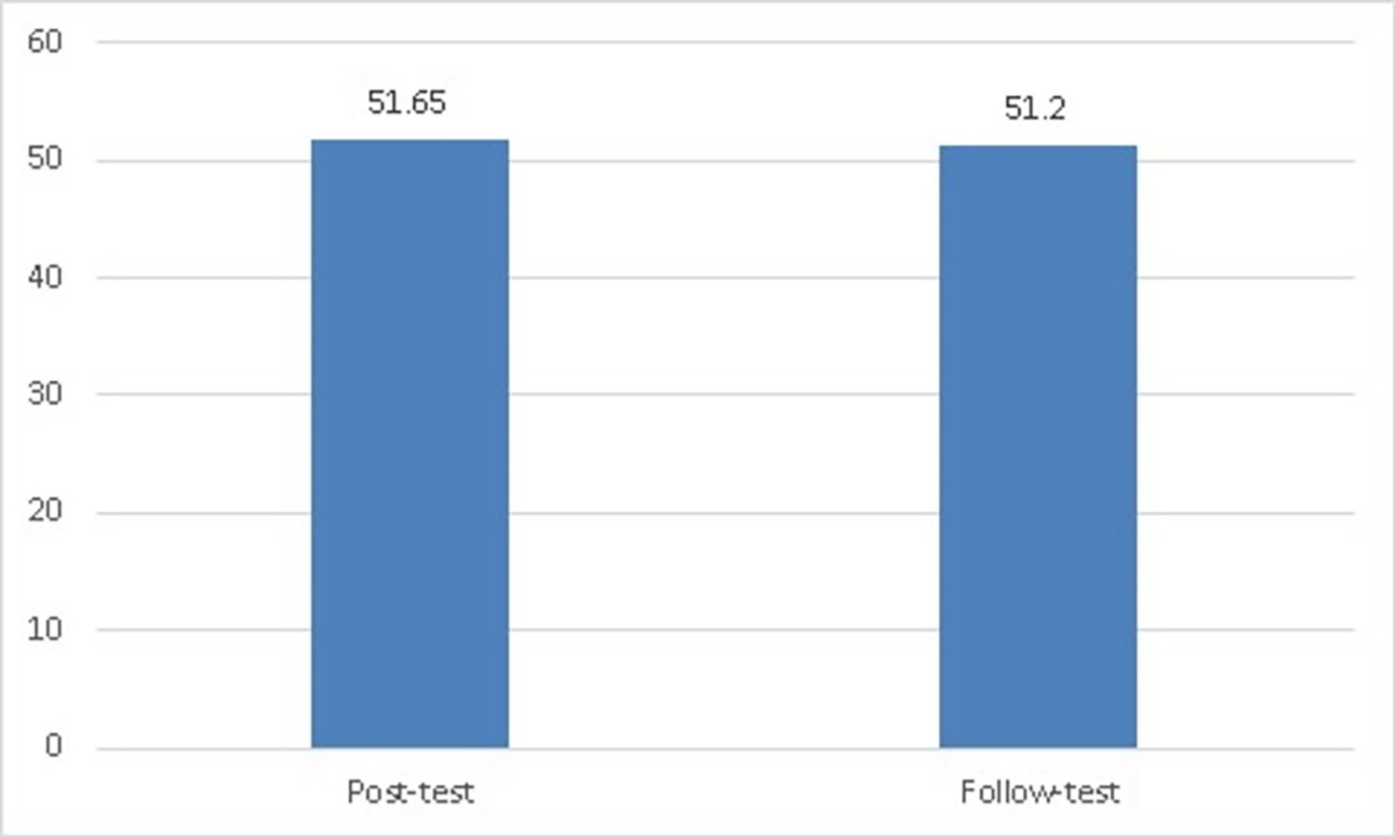
The significant differences between the post-measurement and the follow-up measurement for the experimental group.

Table 7 shows that the Kolmogorov-Smirnov test indicates that the experimental group’s cognitive achievement scores of physical education teaching styles on post-measurement follow a normal distribution, *D* (20) = 0.12, *p* = 0.20. Moreover, a Kolmogorov-Smirnov test on follow-up measurement also follows a normal distribution, *D* (20) = 0.15, *p* = 0.20. Therefore, we can use a paired samples t-test. For the experimental group’s cognitive achievement of physical education teaching style scores, Table 7 and (Fig 3) show neither the post-measurement (*M* = 51.65, *SD* = 2.92) nor the follow-up measurement after one month (*M* = 51.20, *SD* = 2.74) indicated any significant differences, *t* (19) = 2.01, *p* = .06. We therefore accept this null hypothesis.

**Table 7:** Significant differences, statistically, using paired samples T-tests between the post-measurement and the follow-up measurement for the experimental group in the level of cognitive achievement of teaching physical education styles (n = 20).

Variables	Kolmogorov-Smirnov Test	*M* *SD*	*Paired Samples T-test*	
	*D* *df* *P*		*df* *t*	*p*
Post-measurement	0.12 20 0.20	51.65 2.92	19 2.01	0.06
Follow-up measurement after one month	0.15 20 0.20	51.20 2.74		

### Third hypothesis results

*H*_*0*_: There were no statistically significant differences between the post-measurement and the follow-up measurement for the control group in the level of cognitive achievement of teaching physical education styles.

**Fig 4:**
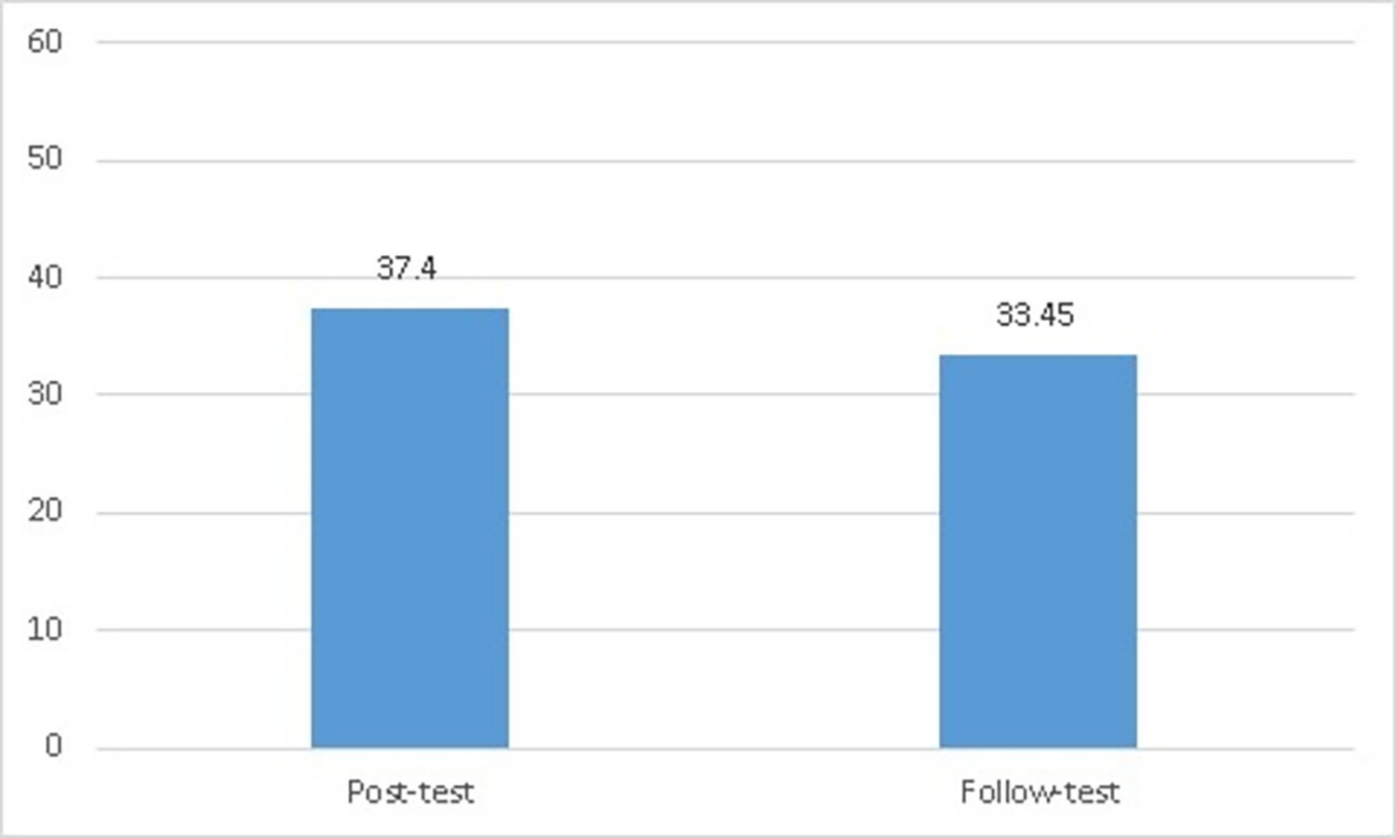
The significant differences between the post-measurement and the follow-up measurement for the control group.

Table 8 shows that the Kolmogorov-Smirnov test indicates that the control group’s cognitive achievement scores of physical education teaching styles on post-measurement follow a normal distribution, *D* (20) = 0.15, *p* = 0.20. Moreover, the Kolmogorov-Smirnov test, on follow-up measurement, also follows a normal distribution, *D* (20) = 0.11, *p* = 0.20. Therefore, we can use a paired samples t-test. For the control group’s cognitive achievement of physical education teaching style scores, Table 8 and (Fig 4) show that the post-measurement (*M* = 37.40, *SD* = 3.51) and the follow-up measurement after one month (*M* = 33.45, *SD* = 3.69) indicated a significant difference, *t* (19) = 9.14, p ≤.05. Consequently, the post-measurement demonstrated significantly better physical education teaching styles than the follow-up measurement. Therefore, the null hypothesis is rejected.

**Table 8:** Significant differences, statistically, using paired samples T-tests between the post-measurement and the follow-up measurement for the control group in the level of cognitive achievement of teaching physical education styles (n = 20).

Variables	Kolmogorov-Smirnov Test	*M* *SD*	*Paired Samples T-test*	
	*D* *df* *P*		*df* *t*	*p*
Post-measurement	0.15 20 0.20	37.40 3.51	19 9.14	0.00
Follow-up measurement after one month	0.11 20 0.20	33.45 3.69		

## Discussion

According to the results of the first hypothesis, the experimental group outperformed the control group on cognitive achievement of physical education teaching style scores. Because of the experimental group students collaborated with and helped one another, they were more likely to learn physical education teaching styles that require high levels of thinking and problem-solving. This is consistent with the findings of Kitchen and McDougall [30], Sharan et al. [61], and Slavin [62], who stated that collaborative learning strategies increase the student academic achievement, interpersonal relationships, awareness of diversity, and ability to think at an advanced level. Moreover, these results are in accordance with those from Johnson [31], Tran [32], Suhendi et al. [33], and Johnson and Johnson [34], in which it is demonstrated that collaborative learning, which was carried out by the experimental group using the Breakout group feature of Blackboard Collaborate Ultra, facilitates the students’ ability to develop knowledge on their own by facilitating their ability to connect with each other. In addition, according to social interdependence theory, the teacher’s efforts to design engaging learning led to positive social interactions within the same group of students, thus raising their interest and making them a more active participant in the educational process, which was a result of the teacher’s efforts to design engaging learning.

Also, the researcher notes that breakout rooms in the Blackboard Collaborate system provide privacy, which makes students feel comfortable in their groups, without feeling embarrassed about making mistakes in front of the teacher. In addition, they share solutions to any problems they face on their worksheets. This is in line with the results of the studies by Oraif and Elyas [63], and Metscher et al. [64], who emphasize the privacy that breakout groups enjoy, which gives psychological comfort to students when they interact.

Moreover, by using breakout groups, students can better collaborate and comment on each other’s work, thus increasing productivity and achieving certain learning objectives, especially because worksheets have already been created that direct students to academic goals, assignments, and time for group work, especially when the teacher is absent; they can also distribute roles among them, such as presenter, dialogue leader, and participant, with an exchange of roles among students. As Read et al. [65], and Yamagata-Lynch et al. [66] described, groups of students must be assigned with more care, certain roles must be assigned to students, and breakout rooms must be more organized. This makes collaborative groups quick, easy, and flexible.

In addition, the researcher suggests that the ease of using the breakout groups allowed the students of the experimental group to save their interactions and send them to the teacher, then present them to colleagues in the main room after the time allotted for the workshops in the breakout groups ended. Furthermore, the breakout groups created a competitive environment between the groups, with each group wanting to display the best work in the main room. The results of Wenzel [67], and Van Heuvelen et al. [68] confirmed that students responded positively to breakout rooms. This was evidenced by the high level of cognitive achievement of the experimental group in comparison to the control group, which relied on an online lecture through a virtual classroom, general discussion, and open-ended questions.

The results also showed that there were no statistically significant differences between the post-test and follow-up test of the experimental group in the level of cognitive achievement after the cessation of any teaching and learning processes for a month. This result consistent with the theory of cognitive load that was explained by Sweller [38], Stiller and Bachmaier [39], de Jong [40], Plass et al. [69], and Stiller [70], which demonstrated that that a learning environment with constructive learning opportunities, positive participation, and cognitive enhancements, such as collaboration, problem-solving, comparison, and classification, contributes to the creation of strong functional mental schemas that promote working memory by enhancing information processing quality and managing it more effectively. This helped the experimental group to maintain its level of cognitive achievement despite the suspension period as compared to the control group, whose level of follow-up measurements decreased due to reliance on virtual classes and lectures.

## Conclusions

This study explored the effectiveness of Blackboard collaborative breakout groups on physical education teaching styles. According to the results, the experimental group achieved greater cognitive achievement than the control group, which received only online lectures through a Blackboard collaborative ultra. In contrast, in the experimental group, breakout groups were formed using collaborative strategies and worksheets. Furthermore, the results showed that the experimental group maintained its cognitive achievement despite the suspension period, compared to the control group, for which their follow-up measurements decreased due to reliance on online lectures. Due to this, it is essential that the online learning process promotes collaboration, engagement, and reinforcement among students. The use of technologies in the classroom must be planned and complemented by teaching strategies with a positive reputation in learning that supports active learning strategies.

## Limitations

Due to the fact that the Physical Education and Kinesiology program at Qassim University is exclusively for males, all participants in this study were male. It would be possible to explore breakout groups in other online courses with female students.

## Implications

The development of more active learning strategies is a very imperative thing that needs to be done to increase students’ engagement and the efficiency of online teaching, so that students will be motivated towards online learning in the future.

## Supporting information

S1 Appendix The cognitive achievement test of the spectrum of physical education teaching styles. (DOCX)

S2 Appendix An example task sheet for organizing experimental breakout groups. (DOCX)
