# Supplementary material for: The impact of Blackboard Collaborate breakout groups on the cognitive achievement of physical education teaching styles during the COVID-19 pandemic
Source: PLoS One. 2023 Jan 6;18(1):e0279921. doi: 10.1371/journal.pone.0279921 (PMC9821457; doi:10.1371/journal.pone.0279921)
Supplement: S1 Appendix — (DOCX) [file pone.0279921.s001.docx]

## Appendix 1

**The Cognitive achievement test of the spectrum of physical education teaching styles**

**Answer Question 1:** Choose the best answer. (48 points)

|  | The axiom in the spectrum of teaching styles in physical education states that teaching behaviour is .................... | | | |
| --- | --- | --- | --- | --- |
|  | - an activity chain | - a chain of styles | - a chain of decision making. | - a chain of lessons |
|  | 2- In direct and indirect styles of teaching physical education, those styles are similar in ...................., and differ in .................... | | | |
|  | - the ratio of decisions – the value | - the value - the ratio of decisions | - the importance – the value | - the value – the importance |
|  | As the purpose of a PE lesson is to develop motor skills such as passing and repeating in basketball while developing social skills, then the following styles are most appropriate...................., | | | |
|  | - The Self-Check and Learner-Initiated Style | - Guided and Divergent Discovery Style | - Inclusion and Learner-Designed Individual Program Style | - Command and reciprocal style |
|  | Three decisions constitute the anatomy of a teaching style: | | | |
|  | - Pre-impact- Impact – Post impact | - Planning - training - explanation | - Direct - indirect - teaching | - Explanation - training - evaluation |
|  | Whenever there are difficulties in implementing a plan, ....................decisions are made | | | |
|  | - Adjustment | - Planning | - Implementation | - Evaluation |
|  | "You are focusing on the ball, so distribute your eyes on the ball and the field". What type of feedback is that? | | | |
|  | - Ambiguous statements | - Neutral statements | - Value statements | - Corrective statements |
|  | As part of the spectrum of teaching styles, ........................................are direct styles | | | |
|  | - Guided and Divergent Discovery Style | - The Self-Check and Learner-Initiated Style | - Command and practice | - reciprocal and Learner-Designed Individual Program Style |
|  | The practice style has the following anatomy: | | | |
|  | - T-L-L | - T-D-O | - T-L-T | - T-L-L_T_ |
|  | The reciprocal style has the following anatomy: | | | |
|  | - T-L-L | - T-D-O | - T-L-T | - T-L-L_T_ |
|  | The inclusion style has the following anatomy: | | | |
|  | - T-L-L | - T-D-O | - T-L-T | - T-L-L_T_ |
|  | ........................................is best for focusing on social development in PE lessons | | | |
|  | - The Command style | - The Reciprocal style | - The Guided discovery style | - The Practice style |
|  | Individual differences between students in PE can be best accommodated by.................... | | | |
|  | - an inclusion style | - the Guided discovery style | - the Command style | - the Practice style |
|  | A lesson that develops the social aspect of the lesson while recognizing the individual differences of the students is most effective when it combines.................... and ....................styles. | | | |
|  | - Guided and Divergent Discovery Style | - The Self-Check and Learner-Initiated Style | - reciprocal and inclusion | - Inclusion and Learner-Designed Individual Program Style |
|  | As an example, basketball free throws are one of the most crucial skills for scoring goals and points: the ....................Style offers the most effective ways to improve them. | | | |
|  | - Command style | - Self-Check | - Guided discovery style | - Convergent Discovery |
|  | As part of the spectrum of teaching styles, ........................................are indirect styles | | | |
|  | - Command and practice | - Self-Check and reciprocal | - Reciprocal and inclusion | - Guided and Divergent Discovery |
|  | The guided discovery style has the following anatomy: | | | |
|  | - T-L-L_T_ | - T-T_L_-T_L_ | - T-L-L | - L-L-L |
|  | The divergent discovery style has the following anatomy: | | | |
|  | - T-L-L_T_ | - T-T_L_-T_L_ | - T-L-L | - L-L-L |
|  | The self-teaching style has the following anatomy: | | | |
|  | - T-L-L_T_ | - T-T_L_-T_L_ | - T-L-L | - L-L-L |
|  | .................... is the best style to develop creativity in PE students | | | |
|  | - The Command style | - The Reciprocal style | - The Divergent discovery style | - The Practice style |
|  | ....................allows students to select their own goals, places, times, and tools | | | |
|  | - The Self-Check style | - The Self-Teaching style | - The Guided Discovery style | - The Convergent Discovery style |
|  | The student is first taught single leg stance. The teacher offers multiple ways to perform the skill. As a result, the student is engaged in an intellectual process about how to perform the correct skill by discussing it with the teacher. In this example, the teacher uses.................... | | | |
|  | - the guided discovery style. | - the Self-Check style | - the Divergent Discovery style | - the Reciprocal style |
|  | The style used when the teacher challenges the students to perform the Single Leg Stance skill in new and creative ways and the students are busy applying the skill in creative ways is .................... | | | |
|  | - the Self-Check style | - the Learner-Initiated Style | - the Divergent Discovery style | - the Guided Discovery style. |
|  | As a result of combining the command and practice styles during implementation, the decision anatomy looks like this: .................... | | | |
|  | - T-L-L_T_ | - T-L-L_T_ | - T-T_L_-T | - T-T-L_T_ |
|  | As students repeat the skill of free throwing the ball in basketball according to their teacher's instructions, they use the worksheet individually to develop accuracy and to assess their performance. There are two types of styles used: .................... .................... | | | |
|  | - command and self-check | - Guided and Divergent Discovery Style | - The command and Learner-Initiated Style | - The Self-Teaching and Guided Discovery Style |

**Answer Question 2:** Select the related decisions in list B for each style in list A. (4 points)

| B | A | The Answer |
| --- | --- | --- |
| 1. Identify alternative ways to perform the skill and interact with students to find the best option | 1. The Reciprocal style | e |
| 1. It is up to the student to make all decisions during the planning, implementation, and evaluation processes | 1. The Inclusion style | d |
| 1. Students design their own individual programs based on the general and specific topics determined by the teacher | 1. The Guided Discovery style | a |
| 1. Students of all abilities can participate in these activities | 1. The Self-Teaching | b |
| 1. Receiving and giving feedback to colleagues |  |  |

**Answer Question 3**: Select (True) if the statement is true or (False) if the statement is false. (4 points)

|  | PE lessons can be taught in two or three styles by the teacher | - True | - False |
| --- | --- | --- | --- |
|  | A practice style focuses on reflection and investigation rather than direct action | - True | - False |
|  | Inclusion is one of the indirect creative styles | - True | - False |
|  | In neutral feedback, the teacher encourages the student | - True | - False |

**Answer Question 4:** Organize four tasks correctly according to the teacher's goal when combining guided discovery and practice. (4 points)

| Tasks | Order |
| --- | --- |
| Students are given the task sheet by the teacher and instructed on how to use it | 3 |
| To accommodate students' individual needs, the playground arena is organized into stations with a variety of difficult activities | NA |
| Alternative skills are presented by the teacher and encouraged to be tried by the students, as well as discuss them with them. | 1 |
| Assist students in discovering the correct alternative skill performance | 2 |
| Students are given an opportunity to use the task sheet, and they receive individual feedback from the teacher | 4 |
| The teacher asks the students to use the skill in various situations | NA |

*NA = Not Applicable
