# Supplementary material for: The impact of Blackboard Collaborate breakout groups on the cognitive achievement of physical education teaching styles during the COVID-19 pandemic
Source: PLoS One. 2023 Jan 6;18(1):e0279921. doi: 10.1371/journal.pone.0279921 (PMC9821457; doi:10.1371/journal.pone.0279921)
Supplement: S2 Appendix — (DOCX) [file pone.0279921.s002.docx]

## Appendix 2

**An example task sheet for organizing experimental breakout groups**

| **Name of students in the group: Date : Time: 20min**  ***Identify the role you would like to play within your group**: Dialogue leader, presenter, and participant   \| **Name** \| *** Role** \| \| --- \| --- \| \| **1-** \|  \| \| **2-** \|  \| \| **3-** \|  \| \| **4-** \|  \| \| **5-** \|  \| |
| --- | --- | --- | --- | --- | --- | --- | --- | --- | --- | --- | --- | --- |
| **The title of workshop:** Direct styles: an anatomy and purpose |
| **Learning outcomes :**   1. The student describes the anatomy of a direct style. 2. The student discusses the purpose of using each direct style with examples. 3. The student collaborates with his colleagues to accomplish group tasks. |
| **Message to the group:**  Dear Students, in the following table, list the direct styles in order, explaining their anatomy and purpose with examples for each. Best regards |
| **The following are the group outputs:** |
| \| **No** \| **Direct Styles** \| **The Anatomy** \| **Useful purposes** \| \| --- \| --- \| --- \| --- \| \|  \|  \|  \|  \| \|  \|  \|  \|  \| \|  \|  \|  \|  \| \|  \|  \|  \|  \| \|  \|  \|  \|  \| |
